# Purine Molecular Interactions Determine Anisotropic Shape of Zebrafish Biogenic Crystals

**DOI:** 10.1002/smtd.202401956

**Published:** 2025-08-21

**Authors:** Jannik Rothkegel, Sylvia Kaufmann, Michaela Wilsch‐Bräuninger, Catarina Lopes, Rita Mateus

**Affiliations:** ^1^ Max Planck Institute of Molecular Cell Biology and Genetics 01037 Dresden Germany; ^2^ Cluster of Excellence Physics of Life Technische Universität Dresden 01062 Dresden Germany

**Keywords:** 2D and 3D segmentation, biogenic crystallization, cryoFIB‐SEM, guanine crystals, iridosome, monte carlo simulations, zebrafish

## Abstract

Across phyla, many organisms self‐organize crystals, for functions like vision, pigmentation, and metabolite storage. In zebrafish, a vertebrate known for its crystal‐based color patterns, iridophores concentrate purines in membrane‐bound organelles, the iridosomes. Inside these vesicles, crystals assemble into large, flat, and thin hexagons following unknown mechanisms that defy typical thermodynamic interactions. Here, we investigate the development of zebrafish iridosomal crystals by using live imaging, cryoFIB‐SEM, and novel morphometric analysis pipelines. In doing so, we find that crystal growth predominantly occurs along the *b*‐crystallographic axis, producing their characteristic anisotropic shape. By performing comparative genetic analyses in vivo and reproducing such conditions in silico, we uncover that the zebrafish crystals’ in‐plane hydrogen bond molecular structure is the main determinant for the observed crystal anisotropy. Macroscopically, the *b*‐axis anisotropy is controlled by the ratio of guanine‐to‐hypoxanthine in the iridosome, without affecting the other axes. At the atomic level, the extent of the (100) facet anisotropy depends entirely on the type, number, and strength of molecular H‐bonds within the crystal lattice. Mechanistically, our work shows that purine diversity and availability inside the zebrafish iridosome is key to form an anisotropic crystal lattice, leading to the observed functional crystal shapes.

## Graphical abstract

Many organisms self‐organize crystals for vision, pigmentation, or storage. In zebrafish, iridophores produce anisotropic crystals in iridosomes. Using imaging and genetic tools, crystal growth is shown to favor the *b*‐axis, shaped by guanine‐to‐hypoxanthine ratios and hydrogen bonding resulting thereof. These findings reveal that purine diversity in the iridosome drives crystal anisotropy, explaining zebrafish's distinct crystal structures and their underlying molecular mechanisms.

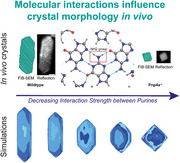

## 1 Introduction

A wide range of organisms utilize organic crystals made of purines, the most abundant of which is the nucleobase guanine,^[^ ^1^, ^2^, ^3^ ^]^ to manipulate light.^[^ ^4^ ^]^ They are used for a multitude of functions, including the production of structural colors,^[^ ^5^, ^6^, ^7^ ^]^ heat protection,^[^ ^8^ ^]^ and even optical components in the visual system.^[^ ^9^ ^]^ Purine crystals can fulfill this plethora of tasks as, depending on their size, morphology, and structural arrangement, they can be used as light scatterers, multilayer reflectors, or image‐forming mirrors.^[^ ^4^, ^10^ ^]^ The very high refractive index of guanine crystals (*n* = 1.83) of the crystallographic (100) plane^[^ ^11^ ^]^ and the fact that guanine is readily available as an end‐product of nucleic acid degradation,^[^ ^12^ ^]^ makes these molecules ideal for building optical elements inside animals efficiently. As crystal morphology is tightly coupled to its cellular function, organisms are capable of robustly influencing and controlling this biocrystallization process. Importantly, disruption of crystal biogenesis undermines organismal fitness and can be detrimental to tissue performance, leading to pathologies.^[^ ^13^, ^14^ ^]^

In vertebrates, guanine crystals are produced by specialized pigment cells called iridophores or leucophores, which differentiate from the neural crest.^[^ ^15^, ^16^ ^]^ These crystals adopt the β‐polymorph structure of guanine,^[^ ^17^ ^]^ composed of hydrogen‐bonded layers, which are stacked via *π*–*π* interactions, leading to diverse prismatic shapes.^[^ ^18^, ^19^ ^]^ In zebrafish (*Danio rerio*), each crystal is intracellularly encapsulated in a membrane‐bound organelle – the iridosome.^[^ ^20^, ^21^, ^22^ ^]^ In this vesicle, crystallization is thought to occur via the accumulation of supersaturated guanine, which undergoes phase transitions into amorphous and then crystalline phases, potentially through the templated nucleation of guanine on preassembled scaffolds.^[^ ^23^ ^]^

Interestingly, recent studies have revealed that biogenic guanine crystals are solid solutions, not only composed of pure guanine, but also including varying amounts of other purines –, e.g., up to 18% hypoxanthine, depending on the organism.^[^ ^2^ ^]^ The finding that biogenic crystals are molecular alloys, has allowed for the rationalization that the presence of small molecules within the crystal lattice can act as dopants, influencing crystal morphology.^[^ ^24^ ^]^ Further, by genetically disrupting Purine nucleoside phosphorylase 4a, i.e., Pnp4a, a crucial teleost protein in the purine metabolic network of iridophores,^[^ ^25^, ^26^, ^27^ ^]^ we have recently shown that reducing guanine production leads to smaller, differently‐shaped crystals, resulting from altered concentration ratios of hypoxanthine‐to‐guanine.^[^ ^25^ ^]^ However, precise quantification of these morphological changes and a detailed understanding of the molecular interactions driving this crystal shape change remain unexplored.

Herein, we use confocal live imaging and electron microscopy techniques to track crystal growth in early zebrafish development from 30 to 96 hours post fertilization (hpf). We demonstrate that biogenic zebrafish crystals preferentially grow along the *b*‐axis, giving rise to their typical elongated shape. By quantitatively comparing crystal morphometrics between wildtype and Pnp4a homozygous mutants, we show that the latter crystals, despite their macroscopic square‐like macromorphologies due to a severely shortened *b*‐axis, have unaltered thickness and width. By performing molecular‐level Monte Carlo simulations, we identify that the observed crystal morphologies in Pnp4a mutants are caused by a disruption of the crystal hydrogen bond network. Finally, by genetically promoting hypoxanthine production in vivo, we show that this manipulation is sufficient to generate iridosomal crystals with smaller *b*‐axis lengths when compared to controls. We suggest that in zebrafish, purine composition at the crystal lattice is instructive for hydrogen bond interaction establishment, consequently regulating (100) crystal facet shape and size. This mechanistic framework can be generalized to explain how crystallographic axis length is regulated in biogenic organic crystals via molecular interactions.

## 2 Results

### 2.1 Morphometric Characterization of Crystal Size and Shape in Zebrafish Iridophores

**Figure 1:**
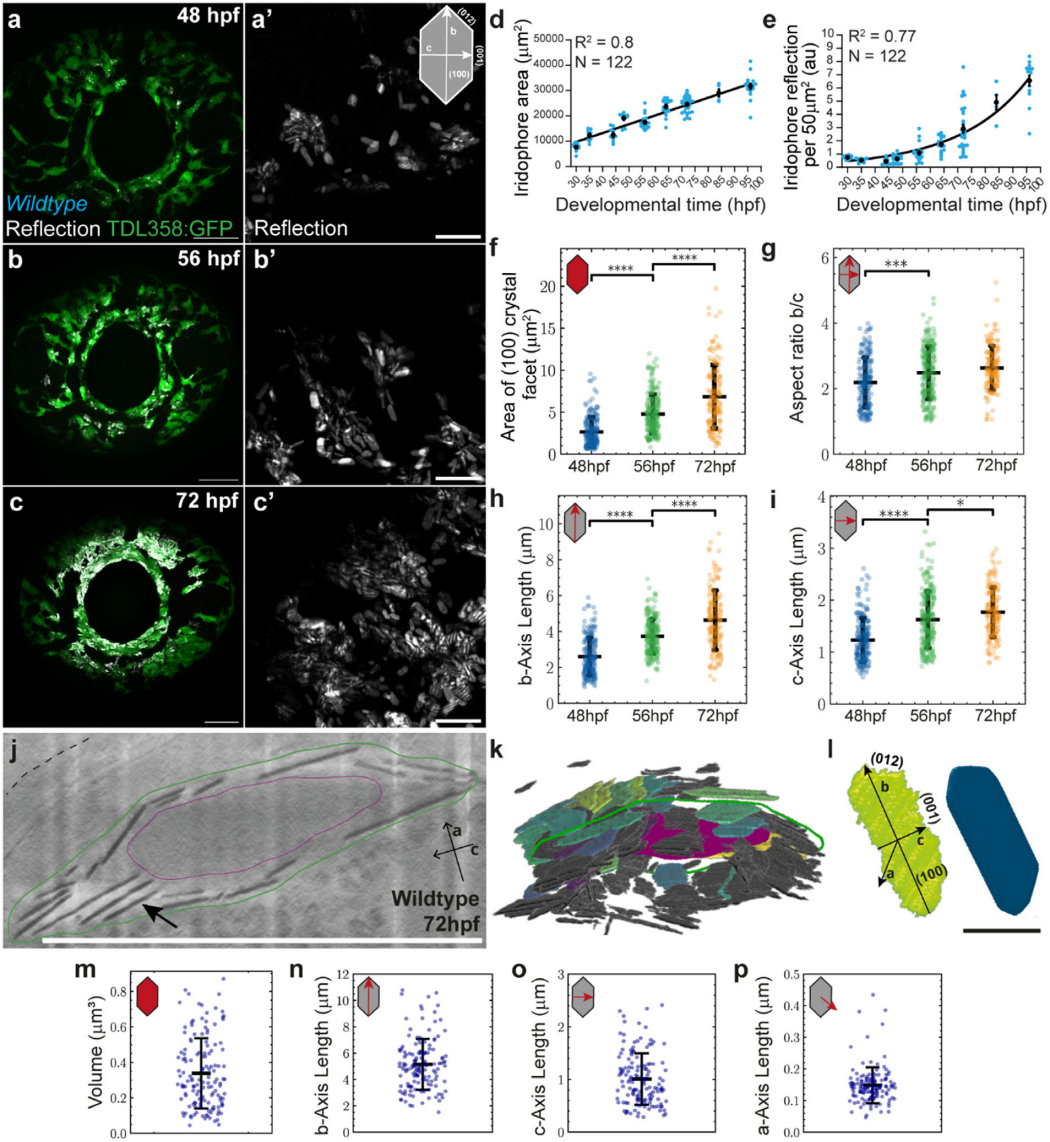
Crystal reflection onset and morphology in larval zebrafish. a–c) Confocal microscopy maximum intensity projection images of larvae eyes of iridophore reporter line (TDL358:GFP, green) and reflection (white), at 48 hpf (a), 56 hpf (b) and 72 hpf (c), with zoom in on crystals (a’‐c’). Cartoon indicates crystallographic axes in 2D with respective crystal facets. Scale bars: a‐c 50 µm, a’‐c’ 10 µm. d,e) Iridophores’ area in the eye (d) increases linearly between 30 and 96 hpf (d; Line, linear fit with goodness of fit, R^2^), while iridophore reflection (e) increases exponentially within the same time interval, in the same cells (e, Line, exponential fit with goodness of fit, R^2^). Mean ±SEM, black lines. f–i) Morphometric measurements from 2D segmented crystals obtained from reflection imaging of wild‐type zebrafish eyes at three different time points. Area of (100) crystal facet (f), Aspect Ratio of *b*‐axis/*c*‐axis lengths (g), *b*‐axis length (h), and *c*‐axis length (i). Asterisks indicate p‐values of unpaired, two‐tailed, non‐parametric t‐tests between groups at each time point: ^∗^ *p* < 0.05, ^∗∗∗^ *p* < 0.001, ^∗∗∗∗^ *p* < 0.0001. Error bars, standard deviation of the mean. N, number of crystals. 48 hpf, *N* = 197; 56 hpf, *N* = 227; 72 hpf, *N* = 107. j) CryoFIB‐SEM slice of wildtype iridophore at 72 hpf. Guanine crystals appear dark due to their high density (black arrow) in comparison to the rest of the cell. The FIB‐milling and imaging direction are parallel to the crystals’ *a*‐axis (inset). Dash, eye surface. Green line, manual annotation of the cell membrane. Magenta line, manual annotation of the cell nucleus. Scale bar, 10 µm. k) 3D crystal reconstruction from (j). Segmented crystals are colored relative to volume, with darker colors corresponding to lower volumes. Cell outline (green) and nucleus (magenta) correspond to the ones depicted in (j). See also Movie S1 (Supporting Information). l) 3D segmentation mask (left) and respective volumetric rendering (right) of a single wildtype crystal (from k), with crystallographic axes and respective facets annotated. Scale bar: 1 µm. m–p) Measurements from 3D segmented crystals obtained from cryoFIB‐SEM imaging; *N* = 139 crystals. Volume (m), *b*‐axis length (n), *c*‐axis length (o), and *a*‐axis length (p).

To elucidate the iridosomal crystal morphology as they develop inside differentiating iridophores, we performed live imaging of transgenic reporter zebrafish that express GFP specifically in iridophores, Et(Ola.Edar:GAL4,14xUAS:GFP)^TDL358Et^ (i.e., TDL358:GFP from here onward).^[^ ^28^ ^]^ We decided to use the larval eye as our developmental model, as this is the first organ that contains iridophores that develop iridosomes, enclosing crystals with stereotypic shapes and sizes beyond the diffraction limit.^[^ ^29^ ^]^ Given the orientation of these vesicular crystals within the cytosol of the iridophores, the planar exposed (100) crystal facets reflect incident light when illuminated, which we took advantage of by using point‐scanning confocal microscopy, in both fluorescent and reflection modes (**Figure** **1**a–c). By quantifying crystal reflection within eye iridophores across time, we found that total eye reflection increases exponentially during iridophore development (Figure 1e). In contrast, the total iridophore area covering the eye increases linearly in time (Figure 1d). Together, this indicates that during this developmental period (30‐96 hpf), the iridophores actively produce large amounts of intracellular crystals, leading to the dense intracellular packing observed with iridosomal guanine crystals (Figure 1c–e).

To extract 2D morphometric features considering the crystal's reflective (100) facet, we developed and implemented a 2D segmentation pipeline to quantify the morphometrics of large numbers of individual crystals in vivo (see Experimental Section). We found that, at 48 hpf, iridosomal crystals have an area of ≈2.63µm^2^, which increases to 4.78µm^2^ at 56 hpf and 6.84µm^2^ at 72 hpf (Figure 1f; Figure S1a, Supporting Information). Interestingly, the contribution of the two crystal axes (b and c) to this increase in area is distinct. The length of the *b*‐axis measures 2.60 µm at 48 hpf and 4.63 µm at 72 hpf, while at the same time, the *c*‐axis length increases from 1.23 to 1.77 µm, showing that the *b*‐axis grows four times faster than the *c*‐axis in a 24 h period (Figure 1h,i). Interestingly, the crystals’ aspect ratio (*b*/*c*‐axis lengths) does not significantly change after 56 hpf, indicating that the crystal's axial proportions are established from this time point onward (Figure 1g). Additionally, we find that regardless of the developmental time point, there is always a population of small crystals (<2µm^2^) (Figure S1a, Supporting Information), indicating that crystal growth is heterogeneous and not fully synchronized per cell. Rather, crystal growth starts individually inside each iridosome, most likely depending on the iridosomal physico‐chemical conditions and the cell's differentiation state.^[^ ^15^, ^16^ ^]^

To increase the accuracy and resolution of our morphometric measurements, we conducted cryogenic Focused Ion Beam Scanning Electron Microscopy (cryoFIB‐SEM) of zebrafish eyes, specifically acquiring images of the iridophores at 72 hpf (Figure 1j; Figure S1d, Supporting Information). This volumetric technique allowed us to directly image high‐pressure frozen samples, where cells and tissues are preserved in their native cellular conditions, including crystal vesicles and the crystals themselves.^[^ ^30^ ^]^ By utilizing cryoFIB‐SEM, we overcame the problematic loss of guanine crystals in developing zebrafish tissues, as conventional EM sample preparation and/or common cellular dissociation and organellar fractionation protocols, prevent the full appreciation of these crystals’ endogenous size and shape.^[^ ^21^, ^22^, ^25^ ^]^ Importantly, this approach also enabled characterization of the crystals’ *a*‐axis (i.e., thickness) quantitatively, given this axis’ nanometer length scale.^[^ ^31^ ^]^ By establishing a semi‐automated 3D segmentation and reconstruction image analysis pipeline, we extracted morphometric measurements of all three crystallographic axes, with respective volumes (Figure 1k,l, Experimental Section). This revealed that crystals have the expected hexagonal morphology,^[^ ^23^ ^]^ independently of the current size (Figure 1k,l; Movie S1, Supporting Information). The crystals display an average volume of 0.34 ± 0.20µm^3^ (Figure 1m), with *b*‐ and *c*‐axis lengths with averages of 5.2 ± 1.93 and 1 ± 0.48 µm for each axis, respectively (Figure 1n,o). By comparing these measurements with our live imaging data, we verified that these are within the standard deviation range of our cryoFIB‐SEM data, which captures such axes in greater detail (compare Figure 1h,i with n,o). The *a*‐axis (140 ± 57 nm) has a narrow length distribution compared to the other axes (Figure 1p; Figure S1c, Supporting Information), suggesting that thickness is tightly regulated within iridosomes.

Taken together, we conclude that when compared to other crystal facets, the (012) facet has a higher growth rate, promoting the expression of the (100) and (001) facets, as the growth rate of a crystallographic facet is inversely proportional to its expression.^[^ ^32^ ^]^ Because of this, the guanine crystals obtain their characteristic hexagonal elongated shape.

### 2.2 The Macroscopic Shape of Zebrafish Crystals is Controlled by Purine Availability in the Iridosome

Recent studies from our lab and colleagues show that the purine composition appears crucial to defining crystal morphology and, consequently, their function.^[^ ^24^, ^25^ ^]^ Given that zebrafish crystals are homogeneous solid solutions composed of not only guanine, but also other purines, namely hypoxanthine,^[^ ^2^, ^25^ ^]^ we hypothesized that the relative amounts of such molecules within the iridophores could significantly affect crystal growth and respective growth directions in vivo.

**Figure 2:**
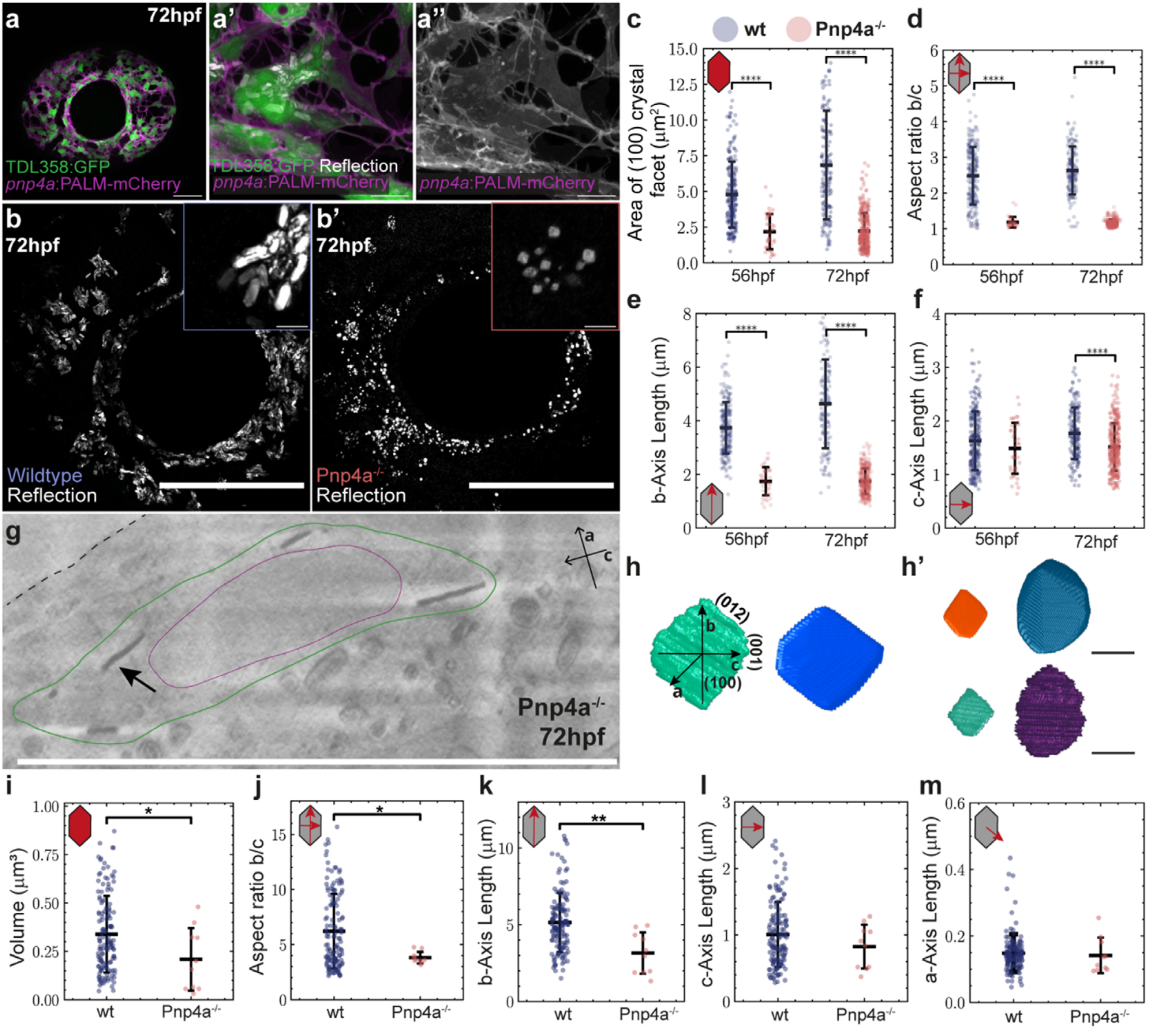
Pnp4a^−/−^ crystals display distinct square‐like macromorphology due to an underdeveloped (001) crystal facet. a) Eye iridophores labeled in double transgenic TDL358:GFP (green) and *pnp4a*:PALM‐mCherry (magenta), with zoom highlighting co‐localization of expression and crystals’ reflection (white), at 72 hpf (a′‐a″). Scale bars: 50 µm (a), 10 µm (a’‐a″). b‐b’ Maximum intensity projection of confocal reflection images of larval eyes in wildtype (b) and Pnp4a^−/‐^ (b’) at 72 hpf. Insets highlight respective crystal morphologies. Scale bars: 100 µm, 5 µm (inset). c–f) Comparisons of 2D morphometric crystal measurements between wildtype (blue) and Pnp4a^−/−^ mutants (red) at 56 and 72 hpf. Area of (100) crystal facet (c), Aspect ratio between lengths of *b*‐ and *c*‐axis (d), *b*‐axis length (e), and *c*‐axis length (f). N, number of crystals. 56 hpf, WT *N* = 227, Pnp4a^−/−^ *N* = 41; 72 2.7kb‐hpf, WT *N* = 107, Pnp4a^−/−^ *N* = 315. g) CryoFIB‐SEM slice of Pnp4a^−/−^ iridophore at 72 hpf. Black arrow, guanine crystal. The FIB‐milling and imaging direction are parallel to the crystals’ *a*‐axis. Dash, eye surface. Green line, manual annotation of the cell membrane. Magenta line, manual annotation of the cell nucleus. Scale bar, 10 µm. h) 3D segmentation mask (left) and respective volumetric rendering (right) of a single Pnp4a^−/−^ crystal, with crystallographic axes and respective facets annotated. h’ While crystals in Pnp4a^−/‐^ mutants express square‐like habits, the extent of their underdeveloped (001) facets presents a range of variability. Scale bars: 1 µm. i–m) Comparison of 3D morphometric measurements between wildtype (blue) (*N* = 139) and Pnp4a^−/−^ crystals (red) (*N* = 10). Volume (i), Aspect ratio of *b*‐axis/*c*‐axis lengths (j), *b*‐axis length (k), *c*‐axis length (l), and *a*‐axis length (m). Asterisks indicate p‐values of unpaired, two‐tailed, non‐parametric t‐tests between groups: ^∗^ *p* < 0.05, ^∗∗^ *p* < 0.01, ^∗∗∗∗^ *p* < 0.0001. Error bars, standard deviation of the mean.

To test the above hypothesis, we resorted to a previously generated zebrafish mutant, *Pnp4a^cbg20^*. In zebrafish, mutating the *purine nucleoside phosphorylase 4a* (*pnp4a*) gene effectively leads to a decrease in cellular guanine concentration, as the Pnp4a protein catalyzes the cleavage of guanosine/deoxyguanosine into guanine.^[^ ^25^, ^27^ ^]^ Despite this, *Pnp4a^cbg20^* homozygous mutants (referred to as Pnp4a^−/−^) were still able to produce crystals, albeit in small amounts, which surprisingly appeared to show a rhomboid‐like habit, due to underexpression of the (001) facets of the hexagonal crystal lattice.^[^ ^25^ ^]^ By performing confocal live imaging of reflection and applying our quantitative image analysis pipelines, we first confirmed that *pnp4a* is highly expressed in iridophores (**Figure** **2**a).^[^ ^33^ ^]^ We then expanded our quantification efforts to compare Pnp4a^−/−−/‐^ crystal morphologies with those of wildtype zebrafish. We observed that crystals produced by Pnp4a^−/−^ mutants exhibit distinct square‐like shapes (Figure 2b‐b’) with an aspect ratio close to 1 (Figure 2d). The mutant crystals are smaller compared to those of wildtype, with an average (100) facet size of 2.20 ± 1.23µm^2^ versus 4.78 ± 2.31µm^2^, at 56 hpf, respectively (Figure 2c). At 72 hpf, Pnp4a^−/−^ crystals do not grow beyond the size already obtained at 56 hpf (2.23 ± 1.26µm^2^, Figure 2c; Figure S1b, Supporting Information), while in wildtype, these reach an average of 6.84 ± 3.80µm^2^ per crystal (Figure 2c; Figure S1a, Supporting Information). The lack of growth of the *b*‐axis in Pnp4a^−/−^ mutants (1.74 ± 0.52 µm at 56 and 72 hpf, Figure 2e) results in the Pnp4a^−/−^ crystals’ morphology phenotype, presenting underdeveloped (001) crystal facets, further validating previous diffraction and in vitro crystallization findings.^[^ ^25^ ^]^

To study crystal morphology in the Pnp4a^−/−^ mutants in more detail, we again conducted cryoFIB‐SEM in zebrafish eye iridophores at 72 hpf. 3D segmentation, reconstruction, and quantification of the acquired cryoFIB‐SEM images, confirmed our initial live imaging observations that the iridophores in Pnp4a^−/−^ mutants produce fewer iridosomes filled with crystals, these being very interspersed within the iridophore cytosol (Figure 2g,h; Figure S1d, Supporting Information).^[^ ^25^ ^]^ The volume of Pnp4a^−/−^ crystals was lower than that of wildtype crystals, with 0.21 ± 0.16µm^3^ (Figure 2i). In line with our light microscopy data and previous work,^[^ ^25^ ^]^ we found that the largest contributor to this volume change is indeed the shorter *b*‐axis, with 3.16 ± 1.35 µm in Pnp4a^−/−^ mutants versus 5.15 ± 1.93 µm in wildtype (Figure 2j,k). Interestingly, the lengths of the a‐ and c‐axes did not change significantly between these two genotypes (Figure 2l,m). Together, these data provide quantitative evidence supporting the notion that a reduced growth rate of the (001) facet in Pnp4a^−/−^ mutants is due to a change in purine levels, resulting in a relative increase in hypoxanthine concentration inside the iridosome. In addition, the Pnp4a mutation also leads to compensatory expression of paralogue genes *pnp5a* and *pnp5b*.^[^ ^25^ ^]^ Interestingly, these proteins have a higher affinity for the catalysis of inosine to hypoxanthine, when compared to the affinity of converting (deoxy)guanosine to guanine.^[^ ^25^ ^]^ This leads to a complex environment occurring within Pnp4a^−/−^ iridophores: on one hand, there's less guanine production, while on the other, hypoxanthine catalysis is favored. This enzymatic milieu is consistent with the possibility of increased hypoxanthine levels within the iridosome, allowing for doping of the crystal lattice, and resulting in the observed shortening of Pnp4a^−/‐^ crystals’ *b*‐axis (Figure 2b,e,h,k).

### 2.3 Zebrafish Crystal Shape is Directed by Bond Type, Number, and Interaction Strength between Purines

Our quantitative results on crystal macromorphology using the Pnp4a^−/−^ mutants reinforce the recently observed connections reporting that molecular crystal morphology depends on crystal purine composition,^[^ ^2^, ^24^ ^]^ specifically when considering the relative purine concentrations within the crystal vesicles.^[^ ^25^ ^]^ Hence, we set out to gain a deeper mechanistic understanding of this relationship by performing molecular simulations of crystal growth. By employing computational quantum chemistry methods and a Monte‐Carlo framework, *Crystal Grower* ^[^ ^34^, ^35^ ^]^ (Experimental Section), we tested how molecular interactions, particularly interactions mimicking hypoxanthine doping in a solid solution scenario, could mechanistically influence zebrafish crystal morphology.

**Figure 3:**
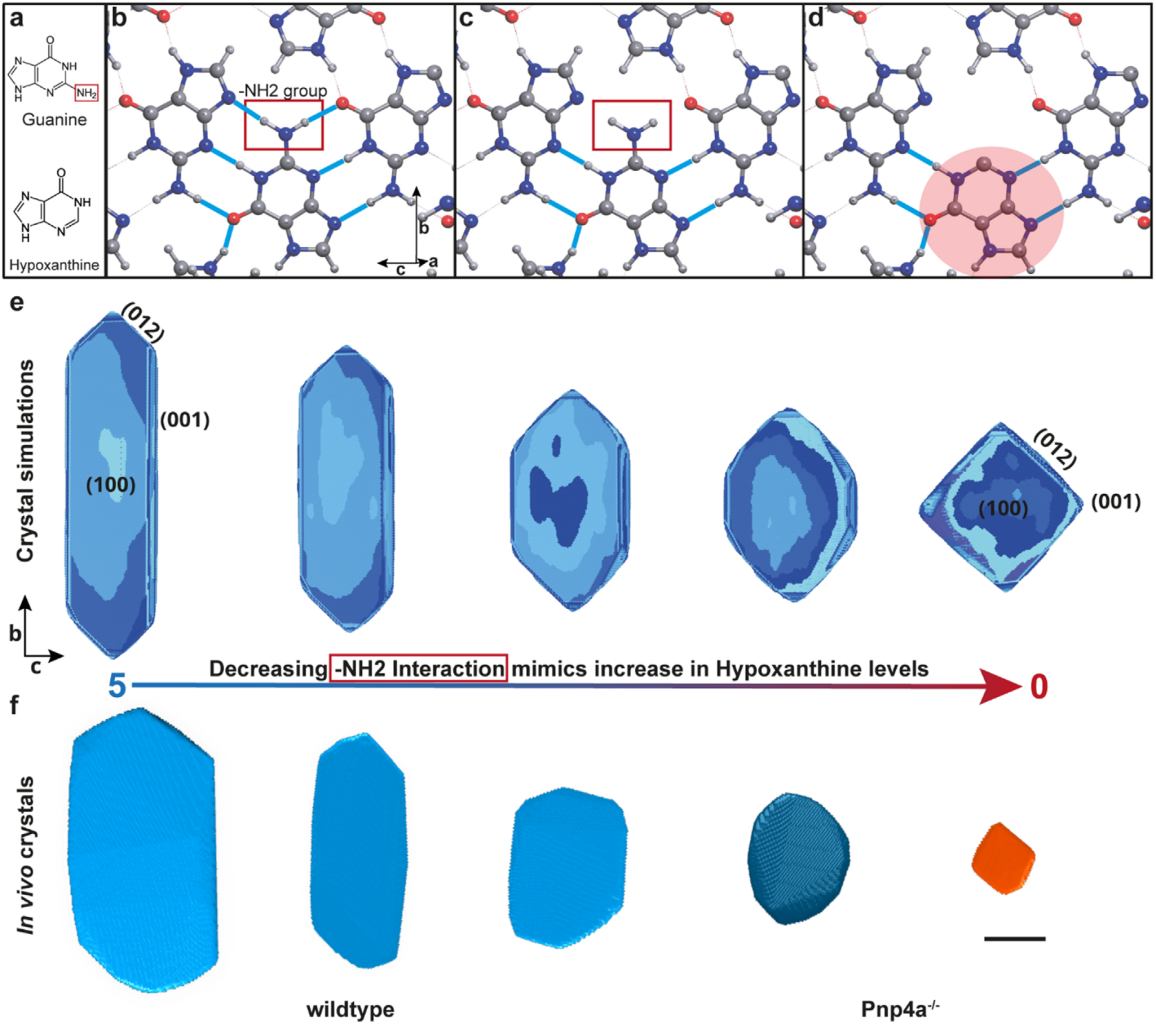
Zebrafish crystal macromorphology depends on H‐bond interaction strength between iridosomal purines. a) Chemical structure of guanine (top) and hypoxanthine (bottom). The two molecules only differ by a ‐NH2 group (red box). b) b‐c plane of the β‐guanine crystal structure. The amino group (red box) forms two hydrogen bonds (cyan) with neighboring guanine molecules. c) b‐c plane of β‐guanine crystal structure without the identified ‐NH2 hydrogen bond interactions (red box in b) corresponds to zero interaction strength in (e). d) b‐c plane of β‐guanine crystal structure with the central guanine molecule being exchanged by hypoxanthine (shaded red molecule). This hypothetical conformation does not have the two hydrogen bonds identified in the pure guanine lattice (red box in b). Like in (c), this corresponds to a scenario where hydrogen bond interactions would have zero cooperation. e) From left to right, simulated crystal morphologies from (c) in decreasing ‐NH2 interaction strength order (range 0–5). Note the striking similarity of simulated crystals with zero interaction strength with the obtained in vivo Pnp4a^−/−^ crystals. f) From left to right, 3D rendered cryoFIB‐SEM crystals aligned with the closest resembling simulated crystal appearance. Scale bar: 1 µm. See also Figure S1e (Supporting Information).

We began by examining the molecular structures of guanine and hypoxanthine, which differ only by the presence of a single amino (‐NH2) group, which is absent in hypoxanthine molecules (**Figure** **3**a). Then, we analyzed the recently refined crystal structure of pure β‐guanine^[^ ^17^ ^]^ by computing the adjacency matrix and determining the types of intermolecular interactions using the *ToposPro* ^[^ ^36^ ^]^ module AutoCN. This network analysis of the guanine crystal lattice identified that the differing ‐NH2 group present solely in guanine is highly interactive with neighboring guanine molecules, forming two hydrogen bonds (Figure 3b compared to 3d). The group forms an additional five Van der Waals interactions, which display weak bond strengths.

In vivo, we observed that Pnp4a^−/−^ crystals present drastic changes in the morphology of the (100) crystal facet when compared to those of wildtype (Figure 2b,c,h). However, the changes in purine composition resulting from the pnp4a mutation do not significantly influence crystal thickness (Figure 2m). Due to this, we focused on understanding within our Monte Carlo framework how can biogenic zebrafish crystals acquire their stereotypic elongated hexagonal shape – a problem effectively reduced to 2‐dimensions. Given that the guanine's ‐NH2 group aligns in its orientation with the crystal's *b*‐axis, which is underexpressed in Pnp4a^−/−^ mutants (Figure 3b), we hypothesized that a higher ratio of incorporated hypoxanthine in the crystal lattice (Figure 3c,d) corresponds to a lower attachment probability on the (012) facet, resulting in the underexpression of the (001) facet. To test this hypothesis, we started by calculating the Voronoi partition of the guanine unit cell with *ToposPro*,^[^ ^36^ ^]^ which yields 12 generalized interactions for each molecule (see Experimental Section, Table S1, Supporting Information). In the *Crystal Grower* framework, each molecule is represented by a proxy atom sitting at its geometric center (Figure S2, Supporting Information), with each generalized interaction passing through one face of the Voronoi polyhedron. We then conducted a parameter search of the 12 identified interactions to determine a parameter space of interaction scalings that resembles the hexagonal crystal morphology found in wildtype zebrafish (Figure 1l; Table S1, Supporting Information, Experimental Section). For simplicity, all crystals were grown under vacuum conditions without considering the energetic cost of desolvation. Remarkably, despite the clear solvent differences to in vivo crystallization settings, we were able to identify a parameter space that produces crystals with striking morphological similarity concerning the biogenic (100) facet (Figure 3e,f, Left; Figure S1e, Supporting Information).

By identifying the generalized interactions that correspond to the hydrogen bonds between the amino group and neighboring guanine molecules (Table S1, Supporting Information), we then tested the influence of the identified ‐NH2 interaction (Figure 3a,b) on the resulting crystal lattice morphology and overall habit. Surprisingly, reducing the strength of this specific interaction in a stepwise manner, while keeping all other interactions unchanged (Figure 3c), leads to the (001) facet being increasingly underexpressed, i.e., shorter, similar to the square‐like macromorphology of the crystals present in Pnp4a^−/−^ mutants (Figure 3e,f, Right). Therefore, we conclude that this ‐NH2 group plays a pivotal role in determining the growth rate of the (012) facet and, consequently, the expression of the (001) facet. From a biological perspective, decreasing the amount of guanine molecules (i.e., Pnp4a^−/−^ mutants), leads to the consequent relative increase in hypoxanthine concentration within the iridosome. In turn, this can function as a doping mechanism of the crystal lattice, as the number and strength of ‐NH2 interactions established at the crystal lattice are directly affected. Consequently, the growth rate of the (012) crystal facet is decreased, as theoretically hypothesized recently.^[^ ^24^ ^]^ In effect, the simple presence of ‐NH2 interactions between guanines in our simulations is sufficient to promote growth along the crystal's *b*‐axis, explaining the phenotypes obtained in vivo with Pnp4a^−/−^ mutants, where such interactions are presumably reduced (given the lower guanine production and enzymatic paralogue compensation), leading to the underexpressed (001) crystal facets (Figures 2b,h and 3e‐f).

### 2.4 Promoting Hypoxanthine Production in Iridophores is Sufficient to Alter Crystal Macromorphology in vivo

Our simulation results point toward a direct relationship between (001) crystal facet expression and the establishment of H‐bonds between adjacent ‐NH2 groups (Figure 3e,f). Given that this amino group is present only in guanine molecules, we hypothesized that varying hypoxanthine levels (as opposed to guanine, as in Pnp4a^−/−^) would also lead to crystal morphologies similar to those previously obtained in our simulations (Figure 3e). To test this hypothesis in vivo, we manipulated the levels of *pnp5a* expression in zebrafish, considering that Pnp5a, unlike Pnp4a, catalyzes inosine in addition to (deoxy)guanosine, producing both hypoxanthine and guanine in zebrafish iridophores.^[^ ^25^ ^]^

**Figure 4:**
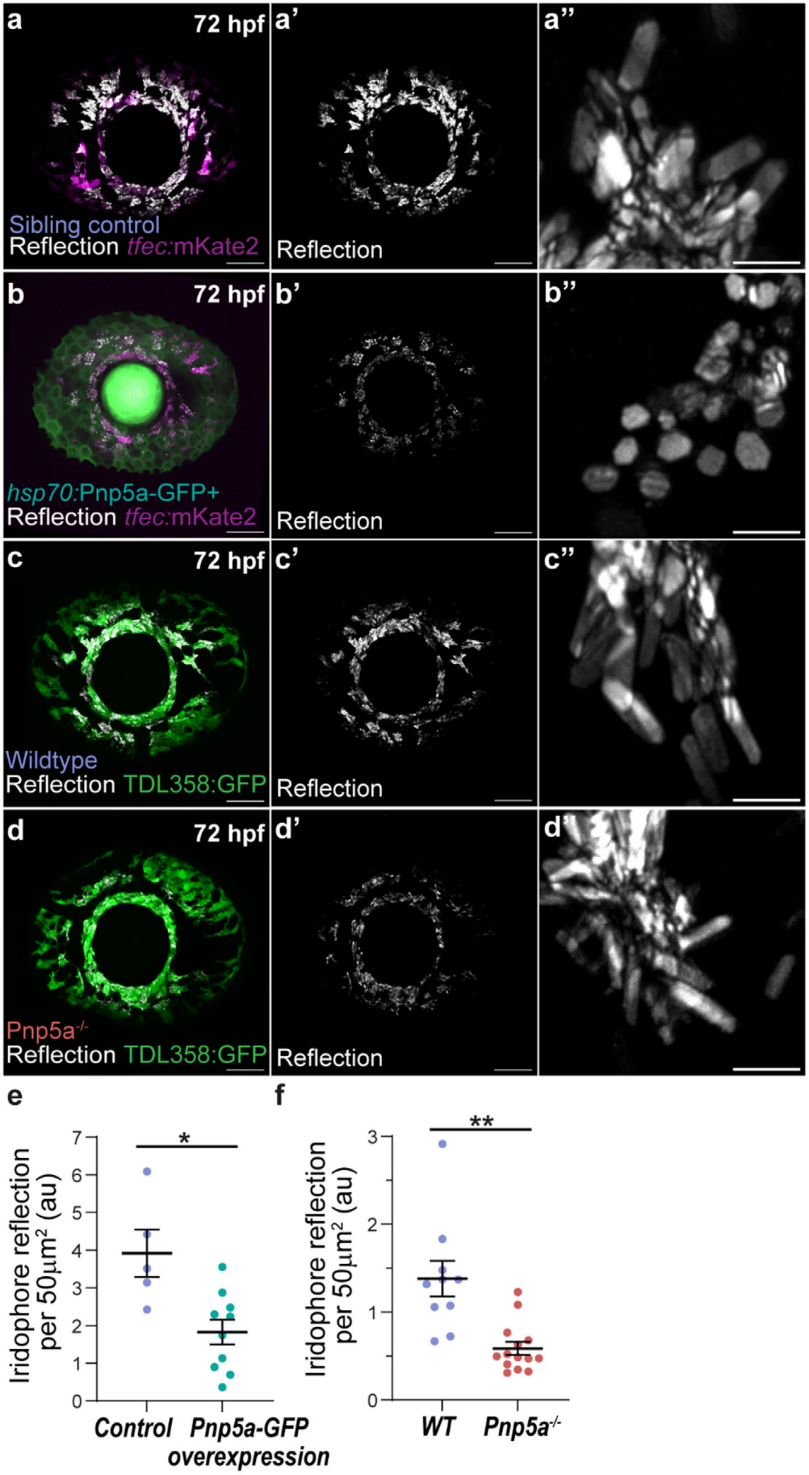
In vivo Pnp5a overexpression leads to smaller (100) crystal facets. a,b) Maximum intensity projections of larval eyes highlighting iridophore reflection upon heat‐shock overactivation of Pnp5a transgene (*hsp70*:Pnp5a‐GFP, green) (b‐b’) or in heat‐shocked sibling controls (a‐a’), at 72 hpf. In both cases, iridophores are labeled by the 2.7kb‐*tfec*:mKate2 (magenta) reporter line. Insets (a″‐b″) highlight respective crystal morphologies. c,d) Maximum intensity projections of larval eyes highlighting iridophore reflection in Pnp5a homozygous mutants (Pnp5a^−/−^, d‐d’) or in wildtype controls (c‐c’), at 72 hpf. In both cases, iridophores are labeled by the TDL358:GFP (green) reporter line. Insets (c″‐d″) highlight respective crystal morphologies. Scale bars: 50 µm (a–d), 5 µm (a″‐d″). e,f) Comparison of the average iridophore reflection per 50 µm in different WT, Pnp5a overexpression (e) or mutant conditions (f), at 72 hpf. au, arbitrary units. Mean ±SEM are shown in black. Heat‐shock sibling control, N = 5; Pnp5a overexpression, N = 10; WT, N = 10; Pnp5a^−/−^, N = 15. Asterisks indicate *p*‐values of two‐tailed, non‐parametric Welch *t*‐tests between groups: ^∗^ *p* < 0.05, ^∗∗^ *p* < 0.01.

Given the established kinetics, we started by testing whether having more hypoxanthine available at the cellular level would lead to crystals with smaller *b*‐axis lengths than wildtype. For this, we engineered a transgenic zebrafish that overexpresses Pnp5a under the conditional control of a heat‐shock promoter (i.e., *hsp70*:Pnp5a‐GFP, Experimental Section). Strikingly, by overexpressing Pnp5a at defined timepoints over the course of iridophore development (Experimental Section), we observed that this was sufficient to generate iridosomal crystals with shorter *b*‐axis (**Figure** **4**a,b). The resulting crystals presented smaller (100) crystal facets, with many showing square‐like macromorphologies, when compared to negative sibling controls (Figure 4a,b). Remarkably, these Pnp5a overexpressing crystals not only resembled the simulations where less ‐NH2 interactions occurred, but also those produced by Pnp4a^−/−^ mutants (compare Figure 4b″ with Figures 2b and 3e,f). Conversely, to study the effects of lack of functional Pnp5a in vivo, we generated biallelic Pnp5a CRISPR mutants (i.e., Pnp5a^−/−^, Experimental Section). Here, we did not observe a change in crystal morphology when compared to wildtype (Figure 4c,d), a phenotype also reported in transient manipulations of Pnp5a in developing eye iridophores.^[^ ^25^ ^]^ In addition to the crystal morphology changes, overall iridophore crystal reflection was reduced in both of these genetic manipulations (Pnp5a overexpression and Pnp5a^−/−^, Figure 4e,f), without affecting the total eye iridophore area (Figure S3a,b, Supporting Information).

To rationalize the obtained phenotypes, we further analyzed the expression of *pnp4a* and *pnp5a* in iridophores along the first five days of zebrafish development, resorting to publicly available single cell RNA sequencing datasets.^[^ ^37^, ^38^ ^]^ Interestingly, while *pnp4a* expression is specific within the pigment cell lineage (Figure S3c, Supporting Information), being highly expressed from 24 hpf in iridophores (Figure S3e, Supporting Information), *pnp5a* expression occurs in many embryonic cell types (Figure S3d, Supporting Information), and in iridophores, its expression is maintained at low levels from 48 hpf to 5dpf (Figure S3e, Supporting Information).

Together, our perturbation experiments and expression analysis explain how zebrafish iridosomal crystals obtain their anisotropic hexagonal morphology: on one hand, the developing eye iridophores do not endogenously express high Pnp5a levels, hence hypoxanthine incorporation into the crystal lattice is minimal; removing it from the system bears no crystal morphology phenotype. On the other hand, Pnp4a is highly expressed in iridophores, consequently guanine molecules are abundant, promoting fast (001) facet expression. Finally, boosting hypoxanthine production by genetic overexpression of Pnp5a, generates crystal lattices that become doped by hypoxanthine; consequently, the probability of guanine‐guanine H‐bond interactions between ‐NH2 groups is decreased, and (001) crystal facet expression is reduced, leading to the observed square‐like macromorphology.

## 3 Conclusion 

### 3.1 Zebrafish Crystal Growth is Uncoupled across Crystallographic Axes

Our study presents the first in vivo comprehensive quantitative analysis of the morphological development of intracellular organic crystals, using zebrafish larval iridophores as a model system. On the one hand, confocal live imaging of reflection identified an active crystal growth phase occurring during iridophore development, where exponential crystal growth and cytosolic packing occur, making these cells functional (Figure 1a–e). On the other hand, this approach allowed us to track the growth of the (100) crystal facet from 48 to 72 hpf. The developed segmentation pipelines enabled precise extraction of 2D and 3D morphological features of single crystals, revealing significant crystal growth along the *b*‐axis, which increased approximately four times faster than the *c*‐axis in wildtype conditions (Figure 1h,i). This differential growth results in the crystals’ characteristic hexagonal, elongated shape. Notably, the aspect ratio between the *b*‐ and *c*‐axis does not change significantly after 56 hpf, indicating that from this developmental time onward, the final proportions of the crystals are set, and crystals increase their size while maintaining their axial proportions (Figure 1g). This points toward tight biological control across crystal shapes, which appears essential in making such crystals functional.

Given the recent reports linking crystal composition and shape,^[^ ^24^, ^25^ ^]^ we explored this connection further in a quantitative mechanistic manner, using a zebrafish line with impaired guanine production, *Pnp4a^cbg20^*. Crystals from homozygous Pnp4a mutants display a distinct square‐like macromorphology with a significantly reduced *b*‐axis length and an almost unaltered *c*‐axis (Figure 2b,e,f). Quantitative live imaging of reflection coupled with cryoFIB‐SEM analyses revealed that Pnp4a^−/−^ mutants produce fewer and smaller crystals than wildtype (Figure 2b,c,i),^[^ ^25^ ^]^ supporting the hypothesis that the molecular composition within the iridosome influences crystal size as well as morphology. While the small crystal size and low numbers seen in Pnp4a^−/−^ iridophores have a profound effect on the reflective properties of these cells (Figure 2 and ^[^ ^25^ ^]^), further investigations probing the efficiency of square‐like versus hexagonal crystal habits will be key to address how form directs subcellular function in this context.

Our findings further indicate that the observed hexagonal shape of the (100) facet (i.e., *b*‐ and *c*‐axis lengths) results from the inherent geometry of the underlying H‐bond network. Nevertheless, when comparing c‐axes between wildtype and Pnp4a^−/−^ crystals, we do not observe significant *c*‐axis length differences (Figure 2l). This suggests that, in Pnp4a^−/−^ mutants, the higher probability of hypoxanthine incorporation into the crystal lattice does not significantly alter the purine binding probability into the (001) facet. Rather, Pnp4a^−/−^ crystals have a disrupted H‐bond network geometry due to the missing amino group, disfavoring the (012) facet but leaving the (001) facet unperturbed.

Concerning the regulation of crystal thickness, i.e., the *a*‐axis, our work indicates that its length is also independently controlled from the other crystallographic axes, as recently also suggested by colleagues.^[^ ^2^, ^24^ ^]^ Importantly, we found no significant differences in crystal thickness between wildtype and Pnp4a^−/−^ iridophores (Figure 2m). Tight control of crystal thickness at the nanometer scale is important in biological systems, as ordering and optical thickness of layers set the efficiency of multilayer reflectors.^[^ ^4^, ^39^ ^]^ This might underlie the differences observed between the average crystal thickness in our dataset from previously reported values in adult zebrafish skin iridosomes.^[^ ^31^ ^]^ The mechanism by which zebrafish and other animals exert control over the thermodynamically favored *π*–*π* stacking direction has so far remained elusive. We deem a stabilization mechanism of crystal thickness based on purine doping unlikely, considering our in vivo analysis of Pnp4a^−/−^ cryoFIB‐SEM data. This idea has also been supported recently by in vitro crystallization and molecular simulation studies.^[^ ^24^ ^]^ In addition, other reports have hypothesized that hydrophobic polymers could stabilize the thickness of the *a*‐axis by preferential absorption on the (100) facet,^[^ ^40^, ^41^ ^]^ or the involvement of templating agents, such as membrane‐bound capping sheets or fibrils.^[^ ^23^, ^42^ ^]^

### 3.2 Crystal Composition Determines *b*‐Axis Length In Vivo

Our simulations of crystal growth using the Monte‐Carlo framework *Crystal Grower* provided new valuable insights into the sub‐molecular interactions shaping crystal morphology. The presence of an amino group in guanine, absent in hypoxanthine, was found to form multiple hydrogen bonds, influencing the growth rate of the (012) facet (Figure 3a,b). Reducing the interaction strength of this ‐NH2 group, simulated an increased doping of hypoxanthine in the β‐guanine crystal structure, which proved sufficient for the underexpression of the (001) facet (Figure 3e). In line with our in vivo results, recent theoretical work supported by in vitro crystallization studies reached similar conclusions.^[^ ^24^ ^]^ In detail, Wagner *et al.* show that synthetically doping the guanine lattice with hypoxanthine disrupts the hydrogen bond network, leading to modulation of the *b*‐axis length in vacuum conditions.^[^ ^24^ ^]^ Interestingly, despite the different approaches taken by our groups, our findings largely agree, and remarkably, this is also consistent with the morphologies observed in wildtype and Pnp4a^−/−^ mutants (Figure 3e,f,^[^ ^25^ ^]^). This suggests that the morphology of the (100) facet in zebrafish purine crystals might only be weakly influenced by solvent conditions, highlighting that our simplified computational model is sufficient to rationalize our in vivo findings.

Altogether, this leads to a unified mechanistic idea that the length of the *b*‐crystallographic axis in biogenic crystals is mainly determined by the molecular interactions established between the purines composing the crystal lattice. Such composition appears controlled by the relative concentration of a variety of crystallized purines inside the iridosome, as evidenced by our Pnp5a overexpression experiments (Figure 4). While here, we show that there is a direct causal relationship between *b*‐axis length and the hypoxanthine doping levels in the crystal lattice in zebrafish, it remains elusive whether this mechanism can be extrapolated to other species.^[^ ^2^ ^]^ Future studies in other purine crystal‐producing organisms, assessing the molecular nature of crystal producing cells and vesicles, coupled with direct functional perturbations of the respective purine metabolic pathways, will be important to determine whether the described molecular interactions at play in zebrafish can be applied as a general principle of crystal morphogenesis.

How cells control diverse iridosomal purine concentrations to achieve controlled intracellular biocrystallization remains an open avenue in the field. This regulation, likely dynamic, can be done at the level of intracellular transport, through respective transporter binding affinities.^[^ ^43^, ^44^ ^]^ Cell type‐specific, dynamic purine concentration regulation can also be achieved at a higher level by altering particular expression levels of different purine phosphorylases (Figure S3c,e, Supporting Information)^[^ ^25^, ^27^, ^45^ ^]^ – effectively changing purine concentration availability in the cell (Figure 4). Further exploration into the cellular and molecular dynamics underlying organic crystallization across species will provide a general framework to understand the making of functional intracellular crystals.

## 4 Limitations of the Study

Here, we present comprehensive quantitative mechanistic insight into the growth and formation of zebrafish purine crystals. Despite this, reflection datasets at 72 hpf may be limited by overcrowding of crystals within iridophores, making the segmentation of single crystals difficult, hence limiting the detection of smaller crystals and rare crystal shapes. Given the nature of Pnp4a^−/−^ mutants, with low crystal count and density, the corresponding cryoFIB‐SEM datasets suffer from low numbers of measured crystals. This may explain the small changes in volume compared to the wildtype datasets. Despite our results being consistent with previous published works,^[^ ^25^ ^]^ a deeper understanding of the crystal orientation and respective facets by performing electron or X‐ray diffraction studies in each of our datasets would be beneficial to complement our conclusions. Finally, all the unit cell calculations used in simulations were run with the pure β‐guanine structure.^[^ ^17^ ^]^ Hypoxanthine doping was only explored with regard to the missing ‐NH2 group, under the premise that the incorporation of guanine and hypoxanthine into the crystal lattice is the same. Given the similarity between the two molecules, and the X‐ray diffraction findings that incorporation of hypoxanthine only distorts the crystal lattice in the *a*‐axis direction,^[^ ^2^ ^]^ we believe this assumption is reasonable.

## 5 Experimental Section

### Zebrafish Lines and Maintenance

All zebrafish (*Danio rerio*) lines were maintained in a recirculating system with a 14 h/day, 10 h/night cycle at 28 °C. Crosses were performed with 3‐ to 12‐month‐old adults. Embryos were kept in E3 zebrafish embryo medium at 28.5 °C until the desired developmental stage was reached. Some fish were kept at 30 °C to reach the 56 hpf stage at 52 hpf. The transgenic and mutant lines used were: Et(Ola.Edar:GAL4,14xUAS:GFP)^TDL358Et.[^ ^28^ ^]^ denominated TDL358:GFP throughout the paper for simplicity, Tg(pnp4a:PALM‐mCherry)^wprt10Tg [^ ^33^ ^]^ and *Pnp4a*.*^cbg20.^* ^[^ ^25^ ^]^ All lines were kept in the wildtype AB strain background. See also Table 1.

### Genotyping and Phenotyping Pnp4a^cbg20^ Mutants

The *Pnp4a^cbg20^* mutation was kept in heterozygosity owing to this genetic alteration being lethal from juvenile stages onward (approximately from 2 weeks old), when kept in homozygosity. As such, individual imaged larvae and breeding adults were genotyped, to confirm mutation zygosity. Briefly, genomic DNA from individual larvae or fin‐clipped tail fins were extracted using the Kapa Express Extract kit (Kapa Biosystems) according to the manufacturer's protocol. This was followed by performing PCR with KAPA2G Robust HotStart ReadyMix (Kapa Biosystems) with primers surrounding the *pnp4a* mutation region (FWD, 5′‐CAGAATTTGTGCTTGTGTTC‐3′; REV, 5′‐CCTTGTACTGGTGATTGTAATG‐3′). As these are in intronic regions, the amplicons were specific to the mutation and not the transgenes present. Then, PCR products were sent for sequencing. Sequences were analyzed using Snapgene. Owing to the very clear phenotypes in homozygous larvae (lack of eye iridescence at 3 days post‐fertilization), this mutation can also be reliably identified in individual fish at this developmental stage. See also Table 1.

### Pnp5a^cbg28^ CRISPR Mutants

CRISPR mutants were generated as described previously.^[^ ^46^ ^]^ Briefly, transactivating crispr RNAs (crRNAs) were designed for specific loci of the *pnp5a* gene (Ensembl ID: ENSDARG00000078619) using pre‐designed crRNAs (IDT). Several crRNAs were tested for ribonucleoprotein (RNP) mutagenesis and were chosen considering where the start codon is located, as well as high on‐target and low off‐target scores. The most efficient crRNA tested was located on exon 2 of the zebrafish *pnp5a* canonical transcript, with target sequence 5′‐CCAGGTGGCCTTCAACTACCGGG‐3′ (Dr.Cas9.PNP5A.1.AD, IDT). The crRNA was annealed with an equal molar amount of tracrRNA (#1 072 533, IDT) and diluted to 57 µm in Duplex buffer (#11‐01‐03‐01, IDT), generating the single guide RNA (sgRNA). The sgRNA was mixed with the Cas9 protein (Alt‐R S.p. Cas9 Nuclease V3; 1 081 058, IDT; 61 µm stock) in equimolar amounts, generating a 28.5 µm RNP solution. To improve mutagenesis efficiency, the mix was kept overnight at −20 °C before injections the following day. One‐cell stage TDL358:GFP embryos were injected with 1 nl of the RNP mix and grown to adulthood. Founder fish containing frameshift mutations were identified by genotyping the resulting F1 progeny, resulting from outcrosses with WT AB fish. Genotyping was performed as above, with primers: FWD, 5′‐CAGGTACAGTTTCGAGGATTG‐3′ and REV, 5′‐CTGTGAAAAATTCCTGACTCTG‐3′. The cbg28 mutant allele effectively shows a 2 bp deletion in exon 2 (resulting from a 4 bp deletion and 2 bp insertion, TACC > CA), shifting its open reading frame. This leads to an early stop codon, generating a truncated Pnp5a protein of 58 amino acids. See also Table 1.

### Total RNA Isolation, Complementary DNA (cDNA), and mRNA Preparation

Total RNA was extracted from 5 dpf embryos using Trizol reagent (Invitrogen) and treated with Dnase I (Roche) according to the manufacturer's protocol. cDNA was synthesized from 1 mg total RNA using the SuperScript IV First‐Strand Synthesis System (ThermoFisher), following the oligo‐dT manufacturer protocol.

For Tol2 mRNA synthesis, the pCS2FA‐transposase vector^[^ ^47^ ^]^ was linearized with NotI (New England Biolabs) and then in vitro transcription was performed using the SP6 mMESSAGE mMACHINE high‐yield capped RNA transcription kit (AM1340, Ambion), following the manufacturer's protocol. The produced Tol2 mRNA was aliquoted and stored at −80 °C until use.

### Transgenic Lines Generation

Transgenesis was performed using the Tol2 transposon system and Gateway cloning technology.^[^ ^47^, ^48^ ^]^ For Tg(*hsp70*:Pnp5a‐eGFP)^cbg30Tg^ generation, the specific pnp5a cDNA (Ensembl ID: ENSDARG00000078619) was amplified from 5 dpf zebrafish total cDNA together with primers containing attB1 and attB2 sites, FWD 5′‐GGGGACAAGTTTGTACAAAAAAGCAGGCTTAATGTTTCCCGAGAGCAACACTGGGTACAG‐3′; REV 5′‐ GGGGACCACTTTGTACAAGAAAGCTGGGTTGGCGTAGTTGTTGTTGTGCTCC‐3′. The 940 bp amplified product was purified using a PCR clean‐up kit (Promega, A9281), and recombined with pDONOR221 (#208, Tol2 Kit) in a BP reaction (BP Clonase II enzyme mix; Invitrogen, 11 789 020). The resulting middle entry clone, pME_Pnp5a, was then recombined in a LR reaction (LR Clonase II Plus enzyme; Invitrogen, 12 538 120) using p5E‐hsp70 (#222, Tol2 Kit), pDEST‐Tol2pA (R4‐R3) (426, Tol2 Kit) and p3E‐eGFP (#440, Tol2 Kit), to generate the final construct, T2‐Hsp70:Pnp5a‐eGFP.

Regarding the Tg(*2.7kb‐tfec*:mKate2)^cbg29Tg^ line, a previously published 2.7 kb *tfec* promoter region (kindly gifted to us by the laboratory of Brian Link) was used.^[^ ^49^ ^]^ The plasmid p5E‐tfec was recombined in an LR reaction (LR Clonase II Plus enzyme; Invitrogen, #12538120) with pDEST‐Tol2pA(R4‐R3) (#426, Tol2 Kit), pME_mKate2 (gift from the Oates laboratory) and p3E‐polyA (#302, Tol2 Kit), to generate the final construct, T2‐tfec:mKate2_pA.

In both cases, one‐cell stage WT AB embryos were injected with 25 pg of one of the final constructs described above, 25 pg of Tol2 transposase mRNA, and 10% phenol Red. At 2 dpf, injected embryos were screened for eGFP or mKate2 using an Olympus SZX16 fluorescence microscope and subsequently grown to adulthood. Founder fish were identified by screening F1 progeny for fluorescence, resulting from outcrosses with WT AB fish. The Tg(*hsp70*:Pnp5a‐GFP)^cbg30Tg^ line is kept in the background of Tg(*2.7kb‐tfec*:mKate2)^cbg29Tg^, allowing for easy iridophore identification and further quantification. A PV‐820 Pico‐injector (World Precision Instruments) and a Narashige micromanipulator were used for microinjection. See also Table 1.

### Embryo Heat‐Shocks

For heat‐shocks, double transgenic embryos Tg(*hsp70*:Pnp5a‐eGFP)^cbg30Tg^; Tg(*2.7kb‐tfec*:mKate2)^cbg29Tg^ were heat‐activated at 36, 48, and 56 hpf in a water bath at 38 °C for 1 h (petri dishes containing 30 ml of embryo medium), subsequently kept at 28.5 °C, selected for fluorescence, and finally live imaged at 72 hpf for reflection and fluorescence.

### Confocal Live Imaging of Reflection

Live imaging by point‐scanning confocal microscopy was performed in larvae anesthetized in 0.1% MS‐222 (Sigma) diluted in E3 embryo medium, and then mounted laterally on a concave slide (Sigma–Aldrich, BR475505) with 0.5% low‐melting‐point agarose (Sigma A9414) in E3 medium. This maximized the position of one of the larval eyes to be perpendicular to the incident laser. A Zeiss LSM880 Airy upright point‐scanning confocal microscope with a dipping water immersion objective (W Plan‐Apochromat 40x/1NA) was used for all live imaging acquisition. Reflection imaging was performed by taking advantage of the optical properties of the flat zebrafish crystals, illuminating the larvae with a 633 nm incident laser, and adjusting the imaging detector to capture reflection arising from the crystals ±10 nm from the incident laser line. Fluorescence and reflection imaging were acquired sequentially for each sample frame. Live imaging was repeated at least 3 times with different biological replicates per condition and zebrafish line.

### Iridophore Area and Reflection Quantification

After full eye confocal image acquisition, maximum intensity z‐projections (MIPs) of all acquired signals were obtained using Fiji.^[^ ^50^ ^]^ The obtained images from the green or red fluorescent protein channel, corresponding to cytoplasmic signal from the TDL358:GFP+ or tfec:mKate2+ cells (iridophores), were segmented using Ilastik,^[^ ^51^ ^]^ using the pixel classification method and batch mode after an initial training dataset. This provided individual binary masks that labeled the contour of all iridophores in the samples. The mask was used to define the regions of interest (ROIs) within the MIPs of other obtained channels, allowing measurements of the corresponding reflectance within iridophores and iridophore area for each sample using a custom‐made Fiji macro. Individual sample reflection was normalized by dividing the reflectance signal per the corresponding iridophore area, allowing comparisons between samples and fitting. Plotting and fitting were performed using Graphpad Prism.

### High‐Pressure Freezing of Zebrafish Larvae

Staged zebrafish larvae at 56 or 72 hpf were anesthetized and then euthanized with MS‐222 (1%, Sigma). Then, heads were removed from embryos with a scalpel blade. Three heads at a time were transferred with a cut pipette tip into high‐pressure freezing carriers made from aluminum (Leica Microsystems or M. Wohlwend GmbH) with 200 µm depth. To preserve the tissues in cryogenic conditions, the remaining liquid was removed and replaced by 10% (or 20%) dextran40 (MW 40 000 g mol^−1^, Roth) in phosphate buffer (0.1 m) at pH 7.2. The sample volume was closed by a flat carrier (B) wetted by hexadecen (Sigma). The sample was then high‐pressure frozen in a Leica ICE high‐pressure freezer (Leica Microsystems, Vienna), and the frozen samples were stored in liquid nitrogen until further processing (up to 1 month).

### Cryogenic Focused Ion Beam Scanning Electron Microscopy (CryoFIB‐SEM)

High‐pressure frozen fish heads in aluminum carriers were transferred into a pre‐cooled Quorum PP3010 cryo preparation system (Quorum Technologies) and mounted on a Zeiss sample holder under liquid nitrogen. This was then transferred into the aQuilo cryo preparation chamber before transferring onto the pre‐cooled Zeiss crossbeam 550 cryoFIB‐SEM (Carl Zeiss Microscopy, Oberkochen), with a cryo‐stage at −160 °C (−180 °C anti‐contaminator temperature). The sample's surface was scanned at a working distance of 5.1 mm with electrons at 2.0 kV, 50 pA, using the Energy selective Back‐scattered (EsB), InLens secondary, or SESI electron detectors to determine the position of the zebrafish eyes. Subsequently, the sample was moved back into the preparation chamber and sputter‐coated with Platinum at 5 mA for 60 s. Back on the cryo‐stage, the sample was tilted gradually to 54°, and the surface was imaged again for orientation. The samples were oriented so that FIB‐milling and imaging direction were parallel to the crystals’ *a*‐axis (Movie S1, Supporting Information). A 60–80 µm wide region containing the pigmented epithelium of the eye was selected, and a trench was milled with the SmartFIB software (Carl Zeiss) using the 30nA@30 kV or 65nA@30 kV FIB probe current. The cross‐sectioned surface was then polished using a 7nA@30 kV probe current to better visualize the features of interest. The cryoFIB‐SEM volumes were then acquired by slicing (40 µm wide) using a 3nA@30 kV FIB probe current for milling, and a 2.5 kV, 50pA SEM beam for imaging, with slice thickness of 32 or 65 nm. The InLens signal alone, or simultaneously with the EsB signal, was recorded with a tilt correction of 36°. The pixel size of the acquisition was 15.4 nm px^−1^ (or 30 nm px^−1^) in the x/y direction. The image store resolution was 2048 × 1536 pixels, although a reduced area was often selected for scanning, concentrating on the region of interest, speeding up scanning time, and reducing beam damage. The cycle time was kept ≈20 s by a line averaging around *N* = 60 for noise reduction. Stacks of ≈500 slices were acquired where possible.

### 2D Crystal Segmentation, Morphometric Feature Extraction, and Quantification

Using the acquired confocal microscopy reflection images, and given that the zebrafish crystals’ *a*‐axis is well below the diffraction limit,^[^ ^29^, ^31^ ^]^ single‐plane crystal images were treated as an effective 2D system, neglecting the crystal thickness and assuming that the (100) crystal plane is parallel to the image plane. To obtain a large dataset of morphological features in a time‐efficient, mostly automated, and reusable way was challenging, as crystals tend to be close to the diffraction limit in early developmental stages, and in later stages, these are very packed, leading to reflection fringe effects due to thin film interference,^[^ ^39^ ^]^ making them hard to be segmented. Therefore, a novel segmentation pipeline was developed, where regions of interest (ROIs) were first picked, where crystals were well separated and fully visible. After selecting these regions, the images were normalized to their respective 99th percentile, enhancing pixel classification's robustness and ensuring that the classifiers remain reusable across different image datasets.

Next, using the Ilastik^[^ ^51^ ^]^ Pixel Classification tool, which allows for semantic segmentation with a random forest classification, the pixels of the training image dataset were classified as either belonging to a crystal or the background. With the pixel mask generated from this classification, single crystals were identified using a simple thresholding method (threshold set at 0.55). A size filter is then applied to exclude all objects smaller than 200 pixels (≈0.5µm^2^). Following this, the identified objects undergo further classification into “good” and “bad” crystals using the Ilastik object classifier. This step is crucial for excluding overlapping or not fully visible crystals. Specifically, object masks are classified as bad if they are concave or possess sharp edges, as these characteristics likely indicate a cluster of crystals that have been mistakenly identified as a single object. Also, any masks colliding with the image border are excluded from further analysis. The resulting masks are used to extract morphometric features, which can be measured. This was accomplished using the regionprops_table function from the scikit‐image library^[^ ^52^ ^]^ and the label statistics function from the Simpleitk library.^[^ ^53^ ^]^ This approach works well for segmenting 2D crystal images up to 56 hpf. Beyond this time point, however, the cells get overcrowded with crystals, making an object‐based segmentation approach unfeasible. This is primarily due to interference effects and the missing spatial separation of the crystals. The freehand measuring tool in Fiji^[^ ^50^ ^]^ was used to segment the 72 hpf datasets and which were then extracted and quantified as above.

### 3D Segmentation, Morphometric Feature Extraction and Quantification of cryoFIB‐SEM Images

CryoFIB‐SEM image stacks are knowingly hard to segment as charging and curtaining artifacts distort the images, and intensities fluctuate strongly.^[^ ^54^, ^55^ ^]^ Therefore, the image stacks were pre‐processed as previously described,^[^ ^54^ ^]^ before segmenting them with a custom‐trained Universal Network (U‐Net). All steps to prepare and segment the images were done with *Dragonfly*. Further, the milling process between consecutive images can often lead to misalignment in the z‐direction. To address this issue, the Sum Squared Difference (SSD) method and manual alignment were employed to ensure proper Z‐Stack alignment. a 3 × 3 median filter was applied across all slices to reduce noise. Next, to counteract fluctuating intensities, the contrast of the images was enhanced by applying CLAHE (Contrast Limited Adaptive Histogram Equalization).^[^ ^56^ ^]^ A kernel size of 150 and 200 bins each were used, ensuring the contrast enhancement was consistent across the entire dataset. Finally, to eliminate curtaining artifacts that can arise during imaging, *Dragonfly*’s decurtaining tool was utilized. Several ROIs were hand‐segmented into a “Background” and a “Crystal” category to generate ground truth annotations. These ground truth annotations were then used to train a fully connected convolutional neural network (U‐Net) inside *Dragonfly* for automated segmentation of FIB‐SEM image stacks. Segmentation masks were corrected manually to separate crystal objects if needed.

Once the crystal segmentation was complete, custom‐made Python code was generated to extract the lengths of the crystallographic axes (a, b and c). In brief, this was done by fitting an ellipsoid (Least Squares Problem) into each crystal object, by centering the coordinates of each crystal's mask (point cloud around zero), and then calculating the covariance matrix. The lengths of the ellipsoid axes were estimated by extracting the eigenvalues of the covariance matrix. For crystal visualization, individual segmented crystals were subjected to *Dragonfly’*s function “Generate contour meshes from ROIs,” which interpolates slight misalignments of individual cryoFIB‐SEM slices (reflected in the segmentation masks) leading to smooth crystal edges. See also Figure S1e (Supporting Information).

### Statistical Analysis

Statistical comparisons of morphological features were done using the Python library SciPy^[^ ^57^ ^]^ by performing a non‐parametric, unpaired, two‐tailed Mann‐Whitney *t*‐test (stats.ttest_ind) between the different conditions. Statistical experimental details can be found in the respective figure legends. All plots were created using the Python library Seaborn.^[^ ^58^ ^]^

### Monte Carlo Simulations using Crystal Grower

*Crystal Grower* ^[^ ^34^, ^35^ ^]^ is a software that allows for molecular‐scale simulation of crystal morphology. It uses a variant of the MONTY algorithm (Monte Carlo on any crystal surface,^[^ ^59^ ^]^), adapted to 3‐dimensions. The approach is grounded in the Bell‐Evans‐Polyani principle that thermodynamics approximates the Monte Carlo rates for a series of closely related chemical processes (such as crystallization). In the framework, entropy differences between the surface sites of the crystal and the crystal bulk are ignored as they are small for most solids, and transitions allowed are immediate exchanges between the mother solution and the crystal phase. Inside *Crystal Grower*, the mother solution behaves like a continuum, supplying and taking away units of growth. This is entirely governed by the parameter Δµ, which contains differences in free energy, entropy, and volume between the solution and the crystal phase.

To predict and simulate crystal growth using *Crystal Grower*, the raw crystal structure file was first prepared. For this, previously published methodology was followed.^[^ ^34^, ^35^ ^]^ In brief, this entailed dividing the structure into Voronoi polyhedra, which were then used as minimal building blocks to reconstruct the crystal via a Monte Carlo algorithm. For this, a primitive unit cell that containing the molecules of interest was first selected. The β‐polymorph crystal structure for our simulations was used, which was recently refined by 3D electron diffraction.^[^ ^17^ ^]^ Next, the adjacency matrix for the chosen structure was calculated using the AutoCN module in *ToposPro*.^[^ ^36^ ^]^ This step determined the types of interactions between the molecules, namely hydrogen bonds and van der Waals interactions. Following this, the Voronoi partitioning for the molecular crystal was calculated using the ToposPro module ADS. The initial parameters given by the *ToposPro* module ADS were fine‐tuned by running a parameter sweep and picking the interactions which resulted in the anisotropic crystal shapes closely matching the ones seen in wildtype zebrafish (Figure 1i). Based on the obtained adjacency matrix, the Voronoi partitioning calculation involves creating a proxy atom at the centroid of the molecules and combining the intermolecular interactions into generalized interactions between these proxy atoms (Table S1, Supporting Information). In the case, this resulted in 12 interactions, with each interaction corresponding to one side of a 12‐sided Voronoi polyhedron (Figure S2, Supporting Information). Finally, the obtained simplified representation is fed into the Crystal Grower interface, where it can be used to reconstruct the crystal via Monte Carlo simulations.

For all simulations, an initial driving force of Δµ = 100 kJ mol^−1^ at a temperature of T = 28 °C was used. This high driving force ensures initial fast nucleation and crystal growth. However, it affects only the growth rate, not the resulting crystal shape. All crystals were grown for 250 000 (≈50 000 particles) steps. The resulting crystal morphology (Figure 3e) is dependent on the scaling (e.g., the attachment probabilities) of the 12 interactions found to occur between the ‐NH2 group among guanines (Figure S2, **Table** **1**; Table S1, Supporting Information).

**Table 1:** Key Resources Table.

Reagent or resource	Source	Identifier
Chemicals, peptides, and recombinant proteins		
Agarose low melting point	Sigma–Aldrich	39 346‐81‐1
MS‐222	Sigma–Aldrich	886‐86‐2
1‐Hexadecen	Sigma–Aldrich	629‐73‐2
Dextran 40	Roth	9004‐54‐0
Oligonucleotides		
Pnp4a_genotyping primer FWD: 5′‐CAGAATTTGTGCTTGTGTTC‐3′	Deis et al.^[^ ^25^ ^]^	n/a
Pnp4a_genotyping primer REV: 5′‐CCTTGTACTGGTGATTGTAATG‐3′	Deis et al.^[^ ^25^ ^]^	n/a
Pnp5a_genotyping primer FWD: 5′‐CAGGTACAGTTTCGAGGATTG‐3′	This study	n/a
Pnp5a_genotyping primer REV: 5′‐CTGTGAAAAATTCCTGACTCTG‐3′	This study	n/a
Experimental models: Organisms/strains		
Zebrafish: AB wildtype strain	European Zebrafish Resource Center	RRID: ZDB‐GENO‐960809‐7
Zebrafish: Et(Ola. Edar:GAL4,14xUAS:GFP)^TDL358Et^	Fadeev et al.^[^ ^28^ ^]^	RRID: ZDB‐FISH‐150901‐2380
Zebrafish: Tg(pnp4a:PALM‐mCherry)^wprt10Tg^	Lewis et al.^[^ ^33^ ^]^	RRID: ZDB‐FISH‐210414‐18
Zebrafish: Pnp4a^cbg20^	Deis et al.^[^ ^25^ ^]^	RRID: ZDB‐ALT‐230926‐12
Zebrafish: Pnp5a^cbg28^	This study	
Zebrafish: Tg(*2.7kb‐tfec*:mKate2)^cbg29Tg^	This study	
Zebrafish: Tg(*hsp70*:Pnp5a‐eGFP)^cbg30Tg^	This study	
Software and algorithms		
Python 3.9.18	Python Software Foundation	https://www.python.org/
Crystal Grower	Hill et al.^[^ ^35^ ^]^	https://crystalgrower.org/
Topos Pro	Blatov et al.^[^ ^36^ ^]^	https://topospro.com/
Dragonfly 2022.2	Comet Technologies Canada Inc.	https://dragonfly.comet.tech/
Ilastik 1.4.0.post1	Berg et al.^[^ ^51^ ^]^	https://www.ilastik.org/
Scikit‐image 0.21.0	Van der Walt et al.^[^ ^52^ ^]^	https://scikit‐image.org/
Fiji	Schindelin et al.^[^ ^50^ ^]^	https://imagej.net/software/imagej/
SciPy 1.10.1	Virtanen et al.^[^ ^57^ ^]^	https://scipy.org/
Seaborn 0.13.2	Waskom et al.^[^ ^58^ ^]^	https://seaborn.pydata.org/
SimpleITK	Yaniv et al.^[^ ^53^ ^]^	https://simpleitk.org/
Prism 8	GraphPad	http://www.graphpad.com/scientific‐software/prism
Snapgene	SnapGene	www.snapgene.com

### Ethical Statement

This study followed European Union directives (2010/63/EU) and German law, with license #TVV52/2021 – ‘Generierung von Zebrafischlinien zur Untersuchung der Größe und Form von Organen und Organellen‘. Genetic engineering work was carried out in an S1 area (MPI‐CBG, S1‐Labore 4., Az.: 54–8451/103, project leader Rita Mateus), following guidelines according to Section 21, Paragraph 1 of the German Genetic Engineering Act, and within projects 01 and 03 from the Mateus laboratory, according to Section 28 of the German Genetic Engineering Safety Ordinance (GenTSV).

## Conflict of Interest

The authors declare no conflict of interest.

## Author Contributions

J.R. performed most live imaging experiments and data analysis, with the help of S.K., C.L., and R.M.; M.W.‐B. processed samples for cryoFIB‐SEM and performed respective imaging; J.R. wrote code and established the 2D and 3D data analysis pipelines as well as leading all Monte Carlo simulations; J.R. and R.M. conceived the project and prepared the manuscript. All authors read and approved the final version of the manuscript.

## Supporting information



Supporting Information

Supplemental Movie 1

## Data Availability

The data that support the findings of this study are available from the corresponding author upon reasonable request. Custom code for the employed analysis pipelines is available on Zenodo at https://doi.org/10.5281/zenodo.15827374. Further information and requests for animals and reagents should be directed to and will be fulfilled by the corresponding author, Rita Mateus (mateus@mpi‐cbg.de).
