# Supplementary material for: Purine Molecular Interactions Determine Anisotropic Shape of Zebrafish Biogenic Crystals
Source: Small Methods. 2025 Aug 21;9(9):e01956. doi: 10.1002/smtd.202401956 (PMC12464799; doi:10.1002/smtd.202401956)
Supplement: Supplementary file 1 — Supporting Information [file SMTD-9-e01956-s002.pdf]

small
methods

Supporting Information

for *Small Methods*, DOI 10.1002/smtd.202401956

Purine Molecular Interactions Determine Anisotropic Shape of Zebrafish Biogenic Crystals

*Jannik Rothkegel, Sylvia Kaufmann, Michaela Wilsch-Bräuninger, Catarina Lopes and Rita Mateus**

Supporting Information for

Purine Molecular Interactions Determine Anisotropic Shape of Zebrafish Biogenic Crystals

*Jannik Rothkegel, Sylvia Kaufmann, Michaela Wilsch-Bräuninger, Catarina Lopes and Rita Mateus**

*Corresponding author, E-mail: mateus@mpi-cbg.de

Table of contents:

Figures S1 to S3

Supplementary Movie 1 Legend

Table S1

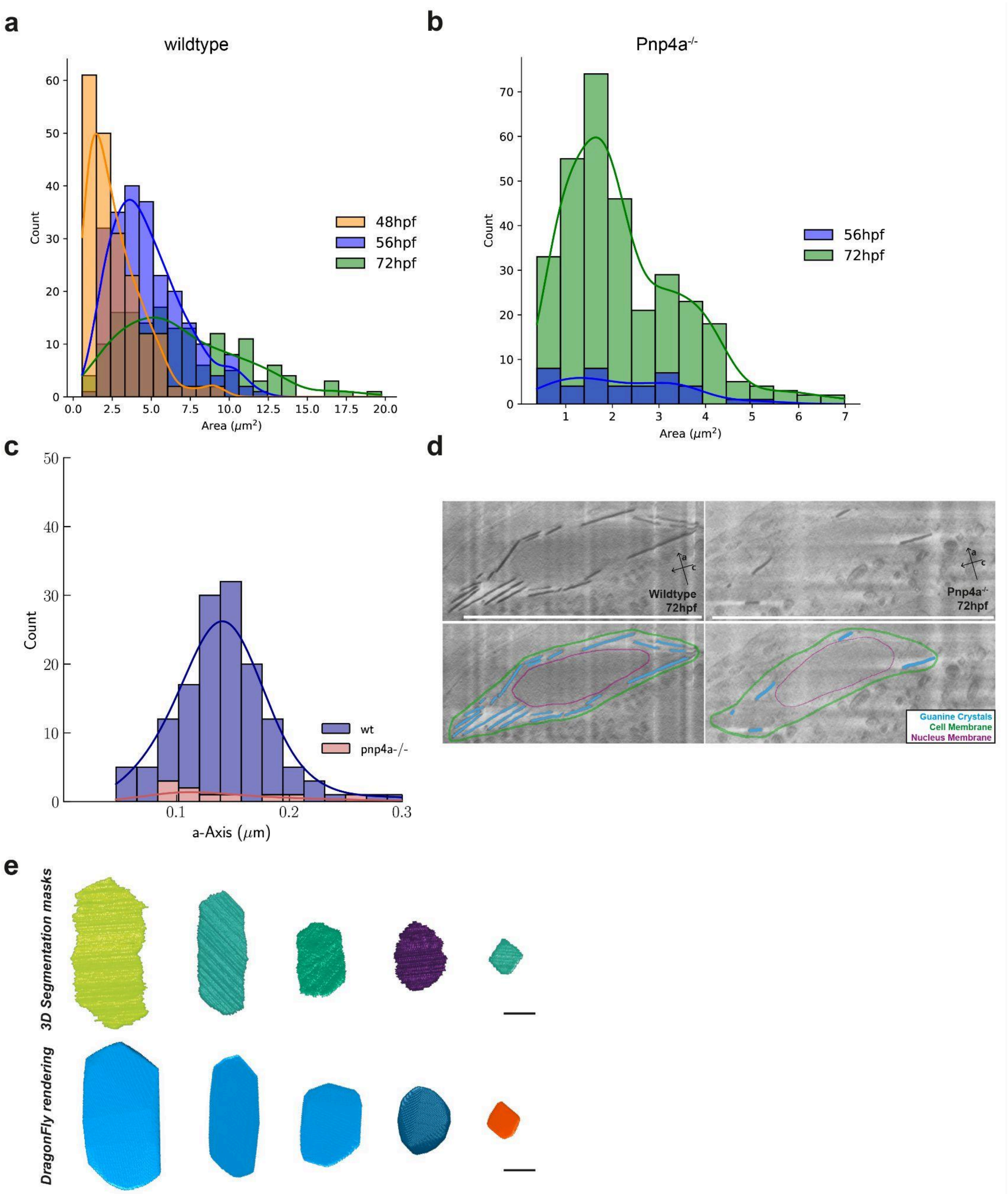

Figure S1. ***In vivo*** **Crystal morphology characterisation.** *(continued on the next page)*

a-b Histograms of (100)-facet area of 2D segmented crystals. Distribution of segmented crystals per area (µm2) at 48 hpf (orange), 56 hpf (blue) and 72 hpf (green), in wildtype (**a**) and Pnp4a$^{-/-}$ mutants (**b**). Lines show kernel density estimation per developmental time point. **c** Histogram of

a-axis length distribution of cryoFIB-SEM 3D segmented crystals, at 72 hpf in wildtype (purple) and Pnp4a$^{-/-}$ mutants (pink). **d** Original cryoFIB-SEM micrographs from Figures 1j and 2g, with corresponding crystal segmentation (blue lines, from DragonFly) and manual annotations (green lines, cell membrane outline. Magenta lines, cell nucleus). Left, wildtype iridophore at 72hpf. Right, Pnp4a-/- iridophore at 72hpf. **e** 3D segmentation masks vs. rendered individual crystals obtained from cryoFIB-SEM images. Scale bars: 1µm.

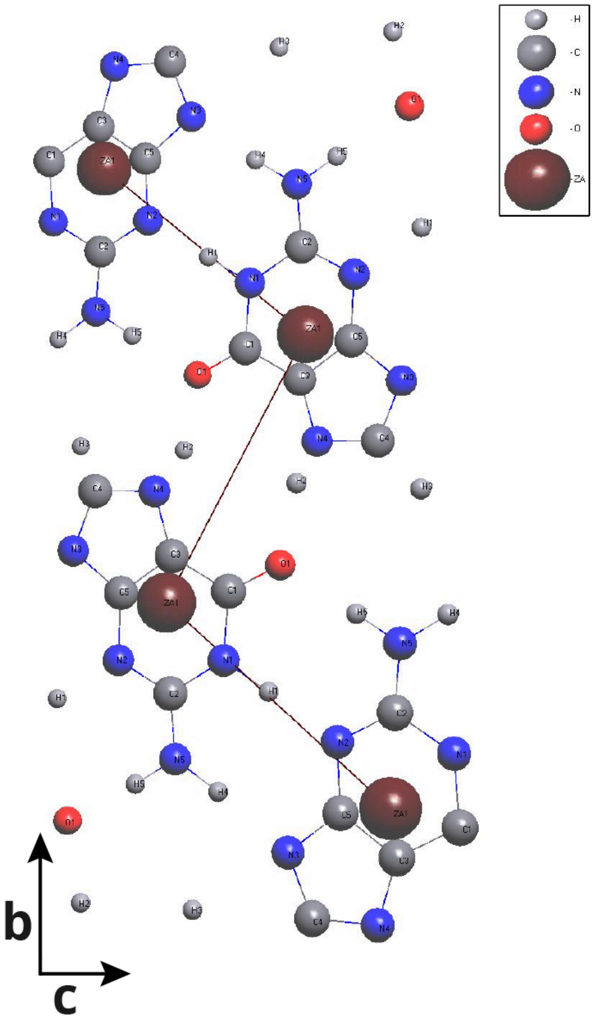

Figure S2. Simulated b-c plane of the guanine crystal lattice. Note that each molecule is represented with a proxy atom (see color legend, top right) at the geometric center of the guanine molecules.

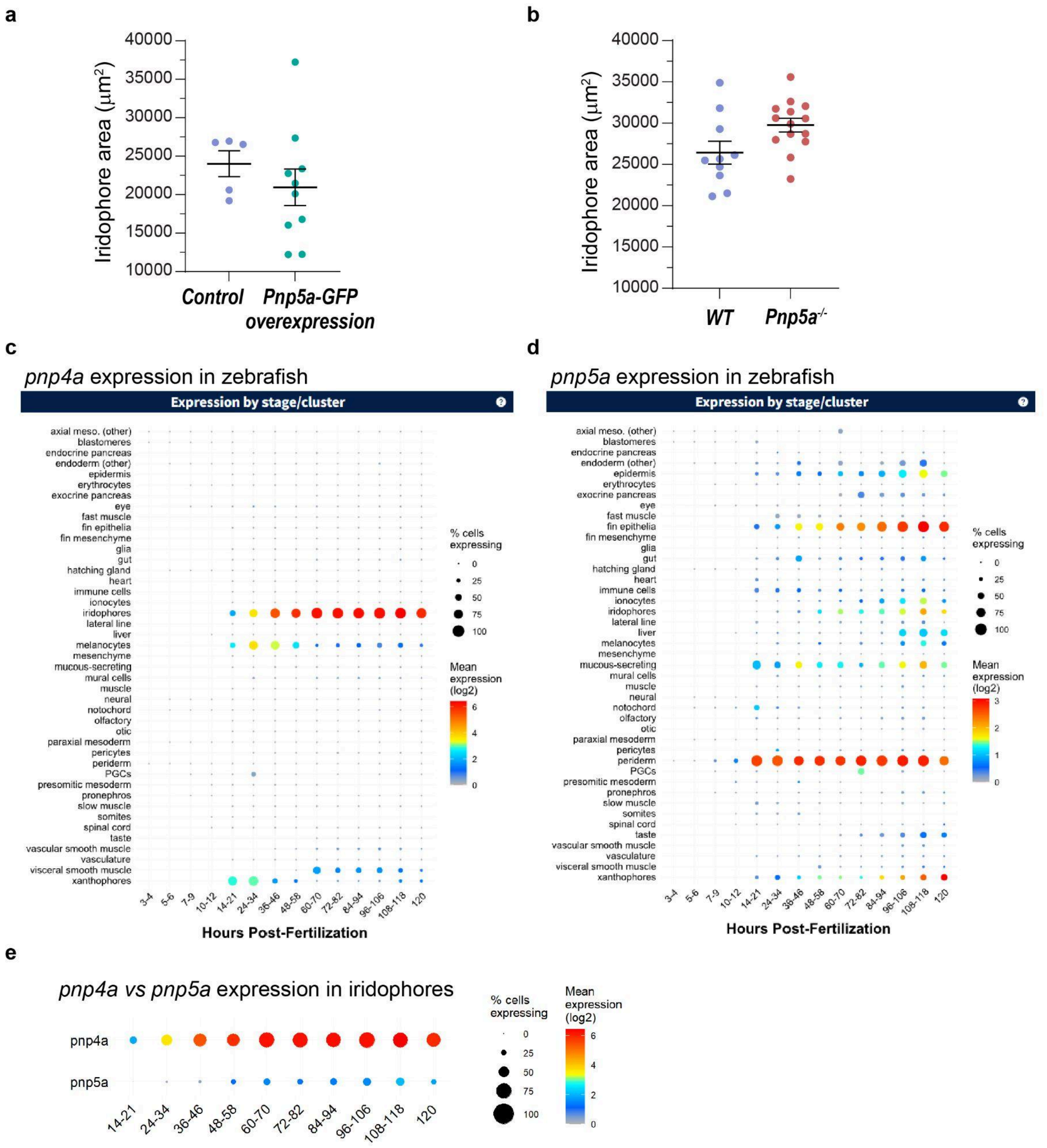

Figure S3. Differential gene expression of *pnp4a* and *pnp5a* during zebrafish development.
(continued on the next page)

a-b Comparison of the average iridophore area per 50µm in different WT, Pnp5a overexpression (**a**) or mutant conditions (**b**), in the 72hpf eye. Mean ±SEM are shown in black. Heat-shock sibling control, N=5; Pnp5a overexpression, N=10; WT, N=10; Pnp5a$^{-/-}$, N=15. Two-tailed, non-parametric Welch t-tests between groups were performed but results were non-significant.

c-d Single cell mRNA sequencing expression values for *pnp4a* (**c**) and *pnp5a* (**d**) between 3 and 120 hpf, in zebrafish embryos. Note that Mean Expression values (log2) are color coded to a different dynamic range for the two genes. **e** Comparison of *pnp4a* vs *pnp5a* expression in zebrafish iridophores between 14 and 120 hpf. Datasets and plots obtained from DanioCell.[36,37]

Movie 1. Cryo FIB-SEM 3D image stack of 72 hpf zebrafish iridophore with reconstructed crystals. Fully segmented crystals are colored relative to volume, with darker colors corresponding to lower volume. The milling direction corresponds to the z-direction, which is parallel to the a-axis.

Name	Space Position	Scaling
A	(-1,0,0)	1
B	(1,0,0)	1
C	(x,1/2-y,1/2+z)(-1,0,0)	1
D	(x,1/2-y,1/2+z)(1,0,1)	1
E	(x,1/2-y,1/2+z)(0,0,1)	3
F	(x,1/2-y,1/2+z)	4
G	(-x,-y,-z)	2
H	(-x,-y,-z)(1,0,0)	2
I	(-x,-y,-z)(2,0,1)	0
J	(-x,-y,-z)(1,0,1)	3
K	(-x,-y,-z)(-1,-1,0)	0 to 5
L	(-x,1/2+y,1/2-z)(1,0,0)	0 to 5

Table S1. Interaction scalings from parameter search.

Interactions in the 1st column (Name) with respective space positions of the proxy atoms, plus interaction scalings used for the simulations in *Crystal Grower*. Green: Van der Waals interaction responsible for pi-pi stacking. Black: Mixed interactions (VdW and H-bonding). Red: H-Bond interactions of the -NH2 residue.
